# Alpha-Linolenic and Linoleic Fatty Acids in the Vegan Diet: Do They Require Dietary Reference Intake/Adequate Intake Special Consideration?

**DOI:** 10.3390/nu11102365

**Published:** 2019-10-04

**Authors:** Bonny Burns-Whitmore, Erik Froyen, Celine Heskey, Temetra Parker, Gregorio San Pablo

**Affiliations:** 1 Nutrition and Food Science Department, Don B Huntley College of Agriculture, California State Polytechnic University, Pomona, CA 91768, USA; ebfroyen@cpp.edu (E.F.); metraous@yahoo.com (T.P.); greggysanpablo@gmail.com (G.S.P.); 2 Nutrition Department, School of Public Health, Loma Linda University, Loma Linda, CA 92354, USA; cheskey@llu.edu

**Keywords:** n-3 fatty acid, n-6 fatty acid, alpha-linolenic acid, ALA, linoleic acid, LA, EPA, DHA, vegans

## Abstract

Good sources of the long-chain n-3 fatty acids, eicosapentaenoic acid (EPA) and docosahexaenoic acid (DHA) include cold-water fish and seafood; however, vegan diets (VGNs) do not include animal-origin foods. Typically, US omnivores obtain enough dietary EPA and DHA, but unless VGNs consume algal n-3 supplements, they rely on endogenous production of long-chain fatty acids. VGN diets have several possible concerns: (1) VGNs have high intakes of linoleic acid (LA) as compared to omnivore/non-vegetarian diets. (2) High intakes of LA competitively interfere with the endogenous conversion of alpha-linolenic acid (ALA) to EPA and DHA. (3) High somatic levels of LA/low ALA indicate a decreased ALA conversion to EPA and DHA. (4) Some, not all VGNs meet the Dietary Reference Intake Adequate Intake (DRI-AI) for dietary ALA and (5) VGN diets are high in fiber, which possibly interferes with fat absorption. Consequently, health professionals and Registered Dietitians/Registered Dietitian Nutritionists working with VGNs need specific essential fatty acid diet guidelines. The purpose of this review was: (1) to suggest that VGNs have a DRI-AI Special Consideration requirement for ALA and LA based on VGN dietary and biochemical indicators of status and (2) to provide suggestions to ensure that VGNs receive adequate intakes of LA and ALA.

## 1. Introduction

The polyunsaturated n-6 and n-3 fatty acids are essential for a variety of functions in the body, including the synthesis of prostaglandins, leukotrienes, cellular membranes, phospholipids, retinal photoreceptors (vision), cerebral gray matter (brain tissue), testes, and sperm. Linoleic (18:2n-6; LA) and arachidonic acids (20:4n-6; AA) are classified as n-6 fatty acids, and α-linolenic (18:3n-3; ALA), eicosapentaenoic (20:5n-3; EPA) and docosahexaenoic acids (22:6n-3; DHA) are n-3 fatty acids [1]. Of these fatty acids, LA and ALA are considered essential and must be provided by the diet.

The Dietary Reference Intakes (DRIs) are “nutrient reference values” developed by the Institute of Medicine of The National Academies. They are intended to serve as a guide for good nutrition and provide the scientific basis for the development of food guidelines in both the United States and Canada, and these nutrient reference values are specified on the basis of age, gender and lifestage groups” [2]. The United States DRIs are used to make informed judgements during the dietary planning process. New research or discoveries of physiological factors, inborn errors of metabolism, health risks, characteristics of the nutrient sources, and nutrient bioavailability may require adjustments in DRI nutrient values for planning individual and group dietary intakes. The present 2003 version of the DRIs states that the nutrients of concern for vegetarians and vegans (VGNs) are vitamin B12, vitamin D, calcium, iron, zinc, and protein [3]. Essential fatty acids in the VGN and vegetarian diets are not presently a DRI concern.

When the DRI concludes that there is inadequate information to set either LA or ALA Estimated Average Requirements (EAR) or the Recommended Dietary Allowance (RDA) for healthy individuals, the Adequate Intake (AI) is used. The present essential fatty acid AI is based on “the highest median intake of LA and ALA in United States adults, where a deficiency is basically nonexistent in non-institutionalized populations” [4]. The present n-6 AI requirement range is 12–11 g/day for men and 17–14 g/day for adult women (non-pregnant/lactating), and the AI n-3 fatty acid recommendations are 1.1 g/day of ALA for women and 1.6 g/day for men [4]. However, it is unknown if the AIs are beneficial or physiologically adequate because dose-response data studies are lacking, Essential fatty acid status is not usually clinically tested, and absence of deficiency symptoms is not necessarily evidence of adequacy.

The authors utilized the definition of ‘vegan’ as the following: a diet which is “devoid of all flesh foods. It excludes eggs and dairy products and may exclude honey” [5]. Frequently, the term ‘strict vegetarian’ or ‘total vegetarian’ is used instead of ‘vegan’ to describe their dietary pattern. Thus, the abbreviation ‘VGN’ is used in this manuscript as a description of the dietary pattern of total vegetarians/strict vegetarians and vegans.

People choose VGN or vegetarian diets for different reasons, which include but are not limited to health reasons, compassion toward animals, a desire to better protect the environment, to lower their risk of chronic diseases, or to therapeutically manage diseases; however, these diets require careful dietary planning. According to the Position Paper of the Academy of Nutrition and Dietetics regarding vegetarian diets, “Well designed vegetarian diets provide adequate nutrient intakes for all stages of the lifecycle and can also be useful in the management of some chronic diseases” [5].

Upon examination of the published articles that relate to ‘vegetarian’, the researcher’s definitions for the terms ‘vegan’ and ‘vegetarian’ are inconsistent, and some researchers consider plant-based diets to be ‘semi-vegetarian’. However, VGN diets are very different from omnivore diets and even diets that are considered to be ‘vegetarian’ (ex. ‘lacto-ovo’, ‘pesco-vegetarian’, ‘plant-based vegetarian’, ‘pollo-vegetarian’, or ‘semi-vegetarian’). These ‘vegetarian’ diets may also include animal-origin foods such as eggs, dairy, fish, or chicken. Thus, dietary patterns and levels of biochemical indicators of essential fatty acid status are very different between VGN and omnivore diets or even other types of vegetarian diets. This narrative review focuses on apparently healthy VGNs and comparisons of VGNs and ‘omnivore’ or ‘meat-eating’ diets, rather than the comparisons of VGN to vegetarian diets or other terms that include the word ‘vegetarian’.

Diet study results between VGNs and omnivores/meat-eaters are inconsistent. Additionally, interpretation or comparison is difficult when some of the information is not reported, or missing. For example, some studies report dietary total polyunsaturated fatty acids (PUFAs), total n-6, total n-3, or collected some, but not all lipid fractions [6,7,8,9,10,11], which limits interpretation of essential fatty acid biological indicators as compared to dietary intakes.

Additional fatty acids of interest are EPA and DHA. They are not considered to be essential since they can be converted from ALA; however, they may be of concern for VGNs, since VGN diets are typically absent of EPA and DHA unless VGNs consume supplements containing EPA and DHA [9,12,13,14,15,16].

Therefore, the purpose of this review was to report and summarize relevant findings of studies on dietary intake and biological indicator status of LA, AA, ALA, EPA, and DHA in plasma, serum, erythrocytes, platelets, breastmilk, and adipose tissue in VGNs, and discuss the reasons why VGN diets may need DRI Special Consideration. We also provide dietary suggestions as to how VGNs can ensure adequate dietary intakes of LA and ALA.

## 2. Methods

### Study Selection

To ensure that peer-review journals were evaluated, the majority of information for articles relating to vegans was accessed from the search engine, PubMed [17] and the searches occurred within the following time frame: 15 June 2017–18 September 2019. The keywords utilized included, ‘vegan’ (827), ‘strict vegetarian’ 179, ‘total vegetarian’ 16, ‘omega-3’ 26183, ‘n-3’ 77245, ‘w-3 fatty acids’ 73, ‘vegetarian’ 4312, ‘omnivore’ 338, ‘alpha-linolenic acid’ 5708, ‘linoleic acid’ 23139, ‘n-6’ (77400), ‘omega-6’ (4964), ‘n-6:n-3 ratio’ 2540, ‘omega-3 index’ (1863), ‘ALA fatty acid’ (2745), ‘ALNA’ (52), ‘LA fatty acid’ (12399), ‘essential fatty acids’ (76261), and combinations of those keywords. Articles were examined to determine if the researchers collected dietary information of VGN and non-vegetarian/omnivore/meat-eater comparisons and/or biochemical indicators of status such as breastmilk, platelet, erythrocyte, plasma, and serum of n-6 and n-3. Additionally, we examined the references in each published article to locate additional articles. We utilized review articles if the reviewer(s) came to original conclusions and/or dietary recommendations based on the studies cited in their review(s).

Studies used in this review regarding comparisons of VGN and non-VGN diets/biochemical indicators are based on adult human and not murine or other animal diet research studies.

## 3. Dietary Sources of the Essential Fatty Acids

The most plentiful dietary n-6 polyunsaturated fatty acid is LA. N-6 fatty acid food sources commonly consumed by VGNs include nuts, seeds, certain vegetables, and vegetables oils such as soybean oil, safflower oil, and corn oil. Therefore, any diet that is mostly plant-based leads to a high dietary intake of LA as well as fiber. Since there are few plant sources high in ALA compared to LA, this may present an intake challenge for VGNs to meet the ALA DRI requirements.

The most abundant n-3 PUFA is ALA, which is found in plant-based oils and/or oil food sources like flaxseed (linseed), English walnut, hemp seed, and chia [18]. The major dietary plant-based sources of LA and ALA are shown in Table 1.

Good sources of EPA and DHA fatty acids are typically found in animals, eggs from laying chickens fed high sources of ALA or DHA, fish or livestock fed n-3 supplements, cold water fish and seafood, or fish which feed on food sources high in ALA, EPA and/or DHA. Some cold water marine algae (except spirulina) contain long chain n-3 fatty acids, and negligible amounts of ALA and LA (Table 1).

Many of the food sources high in LA also contain ALA (Table 1). Thus, it is very difficult to obtain ALA without also increasing the amount of LA in the diet, unless specific foods high in ALA are consumed such as flaxseed, flaxseed/linseed oil, hemp seeds, chia or oils high in high oleate sunflower oil and low in LA (Table 1). Decreasing LA intake in VGNs could be an option; however, decreasing LA intake without monitoring and/or increasing ALA intake might also affect EPA and DHA conversion.

Additionally, there are concerns and considerations such as nutrient bioavailability, physiological implications, and possible health risks of high LA and low ALA in the diet, as well as high LA in plasma, serum, erythrocytes, platelets, breastmilk and adipose tissue.

## 4. Nutrient Bioavailability

It is possible that high fiber diets interfere with fat absorption, including essential fatty acids. Since VGNs typically consume plant products, their diets are typically high in total fiber [6,7,8,14,22]. Soluble fibers such as psyllium, gums and beta-glucan [18], bind fatty acids and prevents them from being absorbed and binds bile, which is necessary for micelle formation. Micelles are required for uptake of long-chain fatty acids into the enterocytes. Insoluble fiber, such as wheat bran, fruit skins, and whole grains [18] increase transit time through the intestine and can also interfere with uptake of fats as well as nutrients in general, because both types of fiber are not digested or absorbed in the small intestine, and humans do not have enzymes to digest fiber. Presently, there are no studies on the effects of high soluble or insoluble fiber on fat digestion in VGNs.

## 5. Physiological Reasons for Special Consideration-Conversion of ALA to EPA and DHA

The essential PUFAs, LA and ALA, must be consumed from the diet, and since VGNs do not consume EPA and DHA from the diet, VGNs rely on endogenous synthesis to obtain long chain n-3 PUFAs. ALA is endogenously converted to EPA and DHA by the elongation and desaturation enzymes in the animal (or marine algae) and becomes incorporated into the animal’s cells and/or stored in adipose tissue. Thus, almost all EPA and DHA dietary sources come from animals and VGNs are limited to algal supplements of EPA and DHA.

Most humans (except those with inborn errors of metabolism) can convert LA to AA and modestly convert ALA to EPA and/or DHA. The human conversion rate of ALA to EPA and DHA is about 5%–8% [23,24,25]. This low conversion rate suggests humans should consume dietary sources of EPA and DHA; however, evidence is not conclusive regarding the amount needed to maintain physiological function.

**Figure 1:**
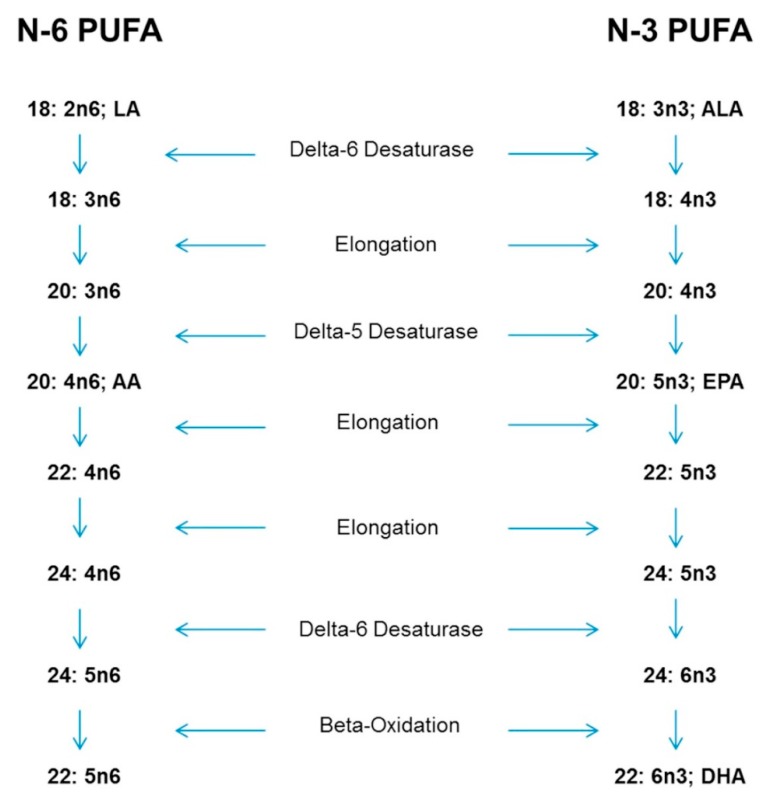
The elongation and desaturation of the essential fatty acids, linoleic acid (LA) and alpha-linolenic acid (ALA). AA: arachidonic acid; EPA: eicosapentaenoic acid; DHA: docosahexaenoic acid; PUFA: polyunsaturated fatty acids. Permission by authors and Elsevier [29].

Both LA and ALA are metabolized by the same biochemical pathway, and excess consumption of LA or ALA results in competition between the elongation and desaturation enzymes for EPA and AA. Excess LA competitively interferes with the ability of ALA to utilize the elongation and desaturase enzymes, thereby suppressing the conversion of ALA to EPA and EPA to DHA [23,24,25,26,27,28] (Figure 1).

Adult and infant studies have demonstrated the conversion of ALA to EPA and DHA through desaturation and elongation, and LA desaturates and elongates to form AA. Through the process of elongation, desaturation and beta oxidation 22:5n-6 forms from AA, using the same enzyme system needed for conversion of EPA to DHA [20,21,22,23,24,25,26,27,28]. In supplemented omnivores and vegetarians, 9.4% of serum phospholipid DHA can be retro-converted to EPA [12].

It may be possible to observe alterations in the synthesis of EPA and DHA from ALA by following a VGN diet over the long-term, as it has been demonstrated that changes in dietary patterns may affect the synthesis of n-3 fatty acids. For example, there is an increased synthesis of DHA from ALA, especially if LA is decreased [30]; ALA is elongated and desaturated in a tissue-dependent manner [31]; and n-3 PUFAs may be depleted during overnutrition [32].

### 5.1. Gender Differences

Studies of premenopausal women reported a higher capacity of ALA conversion, and a more efficient conversion of ALA to EPA and DHA compared to men [23]. In 21 days, women incorporated 700 mg of radioactive labeled [U-13C]-ALA, and resulted in a net fractional ALA interconversion of 21% of EPA, 6% of docosapentaenoic acid (can be converted to DHA), and 9% of DHA in plasma. Over the first 24 h of the study, 22% of the administered [13C]-ALA was recovered as [13C]-CO_2_ on breath analysis, which led the researchers to postulate that increased conversion was due to either an estrogen catalyzed conversion or an increased need for EPA and DHA during pregnancy and fetal development [23].

A similar 24-h study in young men revealed that approximately 33% of administered [U-13C]-ALA was recovered as breath [U-13C]-CO_2_, and the capacity of adult males to convert ALA to DHA was either very low or absent [24]. Due to the low-to-absent conversion in males, the researchers suggested that uptake of pre-formed DHA from the diet may be more “critical for maintaining adequate membrane DHA concentrations in young men” than in women [24,33,34]. Additionally, men tend to convert ALA to saturated fatty acids (SFA) and monounsaturated fatty acids (MUFA) 20% more than women [24]. It is possible that the conversion of the ALA into SFA and MUFA may indicate a reduced capacity for males to convert ALA into the long chain forms, which may result in an additional requirement of dietary EPA and DHA to compensate for a reduced conversion capacity.

### 5.2. Influence of Trans-Fatty Acids on the Δ-6-Desaturase Enzyme

Trans-fatty acids are formed during hydrogenation or in the microbial processes of changing polyunsaturated fatty acids into monounsaturated fatty acids, causing an isomerization of the cis double bond form to the trans double bond form [35]. The main dietary trans-fatty acids are the trans-octadecenoic acids, which contribute to approximately 80%–90% of the total trans-fatty acid content in foods [36]. Trans-fatty acids are found in highly processed foods, baked goods, snack items, candy bars, margarine, and deep-fat fried foods [37]. In addition to lowering HDL cholesterol levels in humans, trans-fatty acids increase lipoprotein (a) levels, raise plasma triglyceride levels, and inhibit the Δ-6-desaturase enzyme, which can interfere with the conversion of ALA to EPA and DHA [38]. This could potentially indicate that persons with a diet high in trans-fatty acids would require increased amounts of ALA and/or EPA and DHA. Therefore, it is important for VGN diet counselors to consider trans-fatty acid intake in VGNs, and researchers to determine trans-fatty acid intake and measure the trans-fatty acid biological status of VGNs. Very few VGN studies reported dietary [8,12] or biological indicators of trans-fatty acid status [12,16,39,40,41].

### 5.3. n-6 Fatty Acid Status

#### 5.3.1. Dietary n-6 Fatty Acid Intakes

Not all studies reported all n-6 fractions intakes, but of the studies that reported n-6 fraction intakes, there were higher LA intakes [8,9,22,40,42,43,44] or non-significant differences of LA intakes [45] in VGNs as compared to omnivores/meat-eaters/non-VGNs. None reported lower LA intakes. Other researchers that measured differences in AA, found lower AA intakes [8,45], or significantly lower in VGNs than omnivores [22,44], and some found no differences in AA intakes [40,42], or did not report AA intakes [9,43].

The present DRI AI recommendations for LA in adults are in the ranges of 17–14 g/day for males and 12–11 g/day for females [4]. Two studies [22,45] indicate that the average VGN LA intake is above recommended intakes, which suggests that VGNs need to decrease intake of LA. However, several other studies did not report LA intakes higher than AI [9,40,42,44].

Most VGN dietary data studies were conducted in countries other than the US [7,9,10,22,40,42,43,44,45], except Rizzo et al. [8] and Sarter et al. [16], and it is unknown whether these food and diet differences can or cannot be applicable or comparable to US VGNs. Therefore, it is prudent to compare the biological indicators of the lipid fraction status between VGNs and non-VGNs to determine the biological storage and/or incorporation differences of the lipid fractions from the diet, and somatic lipids from endogenous conversion.

#### 5.3.2. Plasma n-6 Concentrations

Three studies found significantly higher concentrations of LA in VGN plasma fatty acids than in the meat-eaters, but found no significant differences in AA [10,39,45]. One found no significant differences between plasma LA or AA in either VGNs or omnivores [9] and another reported statistically higher LA and AA plasma choline phospholipids in VGNs as compared to the controls [46] (Table 2).

#### 5.3.3. Serum n-6 Concentrations

Two studies reported significantly higher LA but no significant differences of AA in VGN serum as compared to non-VGN serum [11,42] (Table 2).

#### 5.3.4. Erythrocyte and Whole Blood n-6 Fatty Acid Status

Two studies reported no statistical difference for LA or AA in blood fatty acid profiles of VGNs and meat eaters [9] or soldiers [16]. Three studies reported significantly higher levels of LA in VGNs than omnivores [40,42,46], one found higher AA in omnivore erythrocytes [40], another found high VGN AA in erythrocyte phosphatidylserine [42], and yet another found no significant differences for AA in erythrocytes between VGNs and non-VGNs [46]. No studies showed low levels of n-6 FA in VGNs.

In VGN breastfed infants, LA was significantly higher in the breastfed VGN infant erythrocytes as compared to the omnivore breast-fed infants, and AA was not significantly different from either [42]. Since there were only a few subjects in this study (VGNs *n* = 3, and non-VGNs = 6), more studies are needed.

#### 5.3.5. Platelet n-6 Fatty Acid Status

Li et al. [39], Fisher et al. [47] and Agren et al. [42] reported that VGNs had significantly higher levels of LA in platelets. Both Li et al. [39] and Fisher et al. [47] found significantly lower levels of AA in platelets than the omnivores; however, Agren et al. did not find any AA difference as compared to the omnivores [42].

#### 5.3.6. Breastmilk n-6 Status

Sanders et al. [46] reported significantly higher levels of LA in VGN breastmilk than the omnivore’s, and there were no significant differences for AA. Sanders and Reddy also found a high percentage of LA (23.8%) in breast milk of VGNs as compared to vegetarians (19.7%) and omnivores (10.9%) [48].

#### 5.3.7. Adipose Tissue n-6 Status

Miles et al., found LA was higher in VGNs and lacto-ovo vegetarians (LOVs) than non-vegetarians, and LA was highest in VGNs. AA was significantly lower in VGNs and LOVs than non-vegetarians [41]. Sanders et al. found significantly higher levels of LA in VGN adipose tissue as compared to the omnivore adipose tissue [46].

### 5.4. N-3 Fatty Acid Status

#### 5.4.1. Dietary Intakes of n-3 Fatty Acids in VGNs as Compared to Omnivores

Of the studies that reported lipid diet fractions, some indicate that VGNs consumed high intakes of ALA [12,16,22,40,41,42], lower intakes of ALA [43,44,45], or non-significant differences of ALA [9], as compared to meat-eaters/omnivores. Most studies showed significantly low to zero intakes of DHA [8,9,16,22,40,41,42,43,44,45] and EPA [9,22,43,45] in VGNs unless the VGNs took algal supplements [16] (Table 3).

In some studies, VGN average ALA intake is at, or very close to the AI recommendations for ALA (1.1 in females and 1.6 g/day in males) [45,46]. Kornsteiner et al. [40] and Agren et al. [42] reported high mean intakes of ALA, whereas Pinto et al. reported low intakes of ALA in both omnivores (mean of 1.09 in males and 0.86 in females) and VGNs (mean of 0.71 in females, and 0.84 in males) [9]. In other studies, EPA and DHA VGN intakes were close to 0.00, indicating that dietary intake of EPA and DHA is negligible if not entirely absent in VGNs [9,22,42,44,45], unless the VGN takes EPA/DHA supplements [11].

Mann et al. examined the correlation between dietary intakes and plasma phospholipid concentrations and found that there was a strong significant relationship for all the n-3 fatty acids, as well as AA, indicating that diet contributes to the corresponding fatty acids in the plasma phospholipid concentrations [45]. Agren et al. found that the amount of dietary LA correlated with the proportion of ALA in serum triacylglycerides, cholesterol esters, phospholipids, and in erythrocyte phosphatidylethanolamine; ALA intake did not show any correlations with the n-3 long chain fatty acids, and that the lowest levels of long chain n-3 fatty acids were seen in VGNs with the highest LA intakes, high serum, and membrane lipid levels [42].

#### 5.4.2. Plasma n-3 Concentrations

Rosell et al. [10] reported that plasma ALA was not different between male VGNs and male meat-eaters, whereas EPA and DHA were significantly (*p* < 0.001) lower in the male VGNs than in the male meat-eaters; however, Mann et al. [45], Agren et al. [42], and Li et al. [39] found that ALA plasma was higher in the male VGNs, and EPA and DHA were significantly lower in VGNs. Sanders et al., (1978) did not report ALA values, but AA was significantly higher in VGNs and EPA and DHA were significantly lower [46]. Li et al. reported n-3:n-6 was lower, and AA:EPA was higher than in male high meat eaters [39]. In plasma from females and males, Pinto et al. reported that ALA was higher in VGNs than the high meat group, but EPA and DHA were significantly lower in VGNs, and the values for total trans-fatty acids were not significantly different between the high meat and VGN groups [9]. Conversely, Welch et al. reported that plasma levels for ALA were not different for either males or females as compared to the meat-eaters, but VGN DHA was higher in women than meat-eaters, and in the VGN males, and plasma DHA was significantly lower than in male omnivores [44]. Plasma concentrations of EPA and DHA in female and/or male VGNs were lower than meat eaters or omnivore plasma concentrations in most studies [9,10,39,42,44,45,46]. However, ALA plasma levels were either not different between VGN and non-VGN males [10], or higher [39,42,45] and one study reported that ALA levels were higher in VGN than the meat eater group. One other study found no difference between ALA for either males or female VGNs in comparison to meat-eaters [44].

#### 5.4.3. Serum n-3 Concentrations

Two studies measured serum n-3 concentrations in both VGNs and non-VGNs adults [11,42]. Elorinne et al. reported no statistical difference between VGNs and non-vegetarian serum concentrations for ALA; however, both Elorinne and Agren found that EPA and DHA serum concentrations were significantly lower in the VGNs than the omnivore serum [11,42]. Therefore, in most studies, both serum and plasma levels of EPA and DHA are lower in VGNs than in non-VGNs [9,10,39,42,44,45,46] (Table 3). It is possible that this variability in plasma and serum ALA may reflect short-term ALA status, while the levels of EPA and DHA in plasma and serum may reflect long-term n-3 status in VGNs.

#### 5.4.4. Erythrocyte and Whole Blood n-3 Status

Sanders et al. found that the proportions of DHA in erythrocytes of breast-fed VGN infants was 1.9% as compared to infants fed a milk formula (3.7%). Additionally, they reported significantly high LA, no difference in AA, and significantly low EPA and DHA in VGNs than in the controls [48]. In VGN breast-fed infants, there were significantly lower levels of both EPA and DHA in infant erythrocytes than the breast-fed omnivore infant erythrocytes [46]. In adult erythrocytes, Kornsteiner et al. found similar LA levels in both VGNs and omnivores, and DHA and EPA were markedly decreased in the Austrian VGNs [40]. Agren et al. found that all n-6 proportions were higher in VGNs (except AA), and DHA and EPA were lower in VGN erythrocytes as compared to omnivores, while AA levels differed only in VGN erythrocyte phosphatidylserine, which was higher than omnivores [42]. Kornsteiner et al. suggests that low intakes of long-chain n-3 fatty acids and the unbalanced high n-6:n-3 ratio in VGNs led to substitution of n-3 fatty acids in the membranes, which may be a compensatory change to maintain membrane physical properties [40]. Agren et al. reported differences in the fatty acid composition of sphingomyelin in VGNs as compared to omnivores, which also might be a compensatory change to maintain membrane physical properties [42]. Interestingly, most individuals world-wide have low erythrocyte EPA and DHA concentrations [49], which may increase the risk factors for chronic disease.

Sarter et al. reported significantly higher ALA and EPA levels in VGNs and no differences in DHA levels between the VGNs and the US soldiers in whole blood fatty acids [16]. Since diet comparison data was not reported, it is unknown if these results reflect differences in dietary intakes in the servicemen than in civilian VGNs. However, upon examination of the blood fatty acid profiles, the soldiers’ trans-fatty acid levels were significantly higher than the VGNs, indicating that they consumed more trans-fatty acids than the VGNs, which could explain why the soldiers’ levels of EPA were significantly lower in the whole blood fatty acids. The researchers also reported the baseline blood fatty acids of 166 VGNs and found the omega-3 index was 3.73% with a SD of 1.02%. They compared the omega-3 index values for the soldier and VGN comparison and found that both were 3.5%, which is lower than the Agren et al. study [42], but higher than Sanders et al. study [46].

Pinto et al. determined that the omega-3 index was significantly lower in VGNs (2.71%) than in omnivores (5.42%) [9]. The omega-3 index used by Sarter et al. used the estimated erythrocyte EPA + DHA %weight level and was derived from whole blood DHA + EPA levels by application of a regression equation [16], while Pinto et al. used % weight of EPA and DHA detected with gas chromatography directly from erythrocyte membranes [9]. It is possible that the index results may need to be compared to the same type of methodology, such as comparing calculated with calculated and measured with measured. More studies are needed to determine the reliability, consistency, and sensitivity of each method in determining n-3 status for VGNs.

#### 5.4.5. Platelet n-3 Fatty Acid Status

Li et al. [39] and Sanders and Roshanai [22] found no difference of fatty acid composition of platelet phospholipids between the VGN and high-meat/omnivore groups for ALA; however, Sanders and Roshanai [22] and Agren et al. [42] showed significant decrease in EPA and DHA levels in VGNs. Additionally, Li et al. found a significantly lower concentration of trans-fatty acids in VGNs and a significant difference between n-3:n-6 ratio, and a higher AA:EPA ratio in the VGNs than in the omnivores [39].

Overall, there are few differences of intake of ALA between the VGNs and omnivore/meat-eaters, but there is a consistent, statistically lower concentration/composition difference of EPA and DHA platelet levels in the VGNs compared to omnivores. Even though some VGNs have an ‘adequate intake’ of ALA according to the AI recommendations, it appears the excess LA is interfering with conversion of ALA to EPA and DHA by competing for esterification, suppressing desaturation, increasing production of longer chain n-6 PUFAs, and limiting production of both EPA and DHA in platelets.

#### 5.4.6. Breastmilk n-3 Status

Sanders and Reddy reported levels of ALA breastmilk concentrations comparable to vegetarians but DHA concentrations were less than 50% in both vegetarians and omnivores [48]. Conversely, Sanders et al., (1978) found statistically significantly higher ALA levels in VGNs than the controls, and lower mean values for DHA and EPA, but the values were not statistically significantly different [46]. Perrin, et al. also found that ALA levels were significantly higher in VGNs than omnivores, but DHA and EPA were not significantly different than omnivores. However, 7/26 of VGNs consumed n-3 algal supplements (high in EPA and DHA) as compared to 1/26 omnivores, and this could be a reason for non-significant differences [12].

#### 5.4.7. Adipose Tissue n-3 Status

Few studies utilized adipose tissue for n-3 analyses. Miles, et al. found that ALA was higher in VGNs and lacto-ovo vegetarians (LOVs) than non-vegetarians, with the highest values in VGNs. Docosapentaenoic acid (DPA) and DHA were significantly lower in both VGNs and LOVs, while EPA was also lower in LOVs and VGNs than non-vegetarians. However, total n-3 was highest in VGNs due to high ALA levels [41]. Sanders et al. found that 18:3 n-3 (ALA) was higher in adipose tissue of VGNs than the controls, but it was not significantly different from omnivores [46].

### 5.5. Possible Health Risks of High Dietary LA Intake

Simopoulos noted that LA forms AA and the eicosanoids originating from AA may contribute to inflammatory disorders. Some VGNs consume diets high in n-6 PUFA and diets low in n-3 fatty acids, theoretically increasing cardiovascular disease risk. Thus, in the absence of n-3 derived prostaglandins, thromboxanes and 5-series leukotrienes could cause a physiologic shift to prothrombotic and pro-aggregatory conditions [50]. However, in two diet studies [8,45] AA intake was lower in the VGNs, and Kornsteiner et al. and Agren et al. found no significant differences between omnivores and VGNs for AA [40,42]. In plasma, all the studies that measured AA did not find significant differences between AA level of VGNs and omnivores [9,10,39,46]. Additionally, researchers did not find differences between omnivores and VGNs for AA in serum [9,11,40,42,46], and Sanders and Reddy did not see any significant differences between VGN and omnivore levels of AA in breastmilk [48]. We postulate that these non-significant differences may indicate threshold levels for AA in plasma, serum, erythrocytes, and breastmilk; however, until key lipid products of elongation and desaturation, as well as trans-fatty acid and SFA levels are measured in VGN status studies, we will not know for certain.

Li et al. and Fisher et al. both noted that AA was lower in VGNs in platelets than omnivores, and LA was significantly higher in the VGNs in both studies [39,47]. However, VGNs are at lower risk for cardiovascular disease than omnivores, which may be due to increased phytochemicals in their diet that provide antioxidants and free radical quenchers, and/or lower saturated fat intakes [39,51]. Li et al. [39] noted increased platelet aggregability in VGN males is likely due to low ALA intake. Yu et al. noted that Chinese vegetarians have higher IL-6 and plasma n-6, which may indicate that they are at higher risk for chronic inflammatory diseases [52], and Pinto et al. proposed that more research is needed on low n-3 status and increased risk of a pro-inflammatory profile in VGNs [9].

### 5.6. Comparisons of High LA Biological Levels and n-3 Fatty Acid Status

Comparison studies between high meat/omnivore groups and VGNs indicate some highly significant differences between n-3 and n-6 status of VGNs and omnivores. LA concentrations are high in VGN plasma, platelets, and erythrocyte fatty acids in all studies, except for the male VGNs in the Sarter et al. study [16]. Additionally, the research consistently showed a lower concentration of EPA in VGN tissues as compared to omnivores/meat eaters [9,10,11,22,38,40,42,43,45,46]. Additionally, DHA levels are lower in most studies [9,10,11,22,40,42,43,45,46], except four [12,16,41,44]. Sarter et al. [16] showed higher DHA levels in the VGNs, which were mostly male, while Welsh et al. [44] showed higher DHA status in the five women VGNs. Miles at al. [41] showed no significant difference between groups, and Perrin et al. [12] included 7/26 VGN women that took EPA/DHA supplements, which possibly influenced the outcome(s).

## 6. Recommended Intakes of n-3 and the n-6:n-3 Ratio

Expert opinions differ when it comes to n-3 and n-6:n-3 ratio recommendations (Table 4—most recent studies are listed first) and overall, the recommendations for dietary n-6:n-3 suggested by experts and researchers are lower than 10:1 and between the range of 2–4:1 [40,43,53,54].

The ratio of n-6 fatty acids to n-3 fatty acids can influence the conversion of ALA to EPA and DHA [58,59]. Gerster found that a diet high in n-6 reduces the ALA to EPA and DHA conversion by 40–50% [58]. When healthy men increased dietary intake of LA from 15 g/day to 30 g/day (increased n-6:n-3 ratio) this resulted in a 40–54% increase in conversion of LA to AA, as well as a subsequent decrease in conversion of ALA to EPA and DHA [59]. Additionally, high n-6:n-3 ratios in obesity are directly related to non-alcoholic fatty liver disease [32].

Several VGN studies have determined dietary intake and status of the n-6:n-3 ratios. The majority of VGNs have n-6:n-3 plasma ratios that are ‘unbalanced’ [10,13,22,40,42,53]. Kornsteiner et al. reported that a ratio of 10:1 promoted n-3 tissue declines and reduced desaturation and elongation of n-3 fatty acid-related products, and suggested that VGNs achieve a balanced ratio of n-6:n-3 in their diet to enhance the conversion of ALA into EPA and DHA [40]. A reduction in the n-6:n-3 ratio to 2–4:1 was shown to maintain normal metabolism and increase the amount of long chain PUFAs synthesized in the body [22]. Simopoulos affirms a balanced n-6:n-3 ratio is a determinant of health because n-6 and n-3 fatty acids influence gene expression, and advocates that a balanced ratio of LA to ALA of 2:1. She also stated that the n-6:n-3 ratio of 10:1 in the diet indicated Western diets are deficient in n-3 [54]. Davis and Kris-Etherton suggested optimizing status by consuming double the intake of ALA (≥1% of energy or 1.1 g/1000 Kcals and aim for 2–4 g/ALA/day) and a direct source of DHA in the range of 100–300 mg/day [53]. The authors of the Position Paper of the Academy of Nutrition and Dietetics on Vegetarian Diets suggest that the LA/ALA ratio should not exceed 4:1 and that “it may be prudent to ensure a somewhat higher intake of ALA” [5].

A dietary increase in flaxseed (linseed) oil increases plasma ALA and EPA and lowers the ratio of n-6:n-3 [44,60,61,62]. Li et al. showed dietary ALA from canola and linseed (flaxseed) oil increased the EPA tissue profile 2.5-fold in a high ALA diet compared with a 0.5-fold increase in a moderate ALA diet in male subjects 20 to 50 years old [39]. Indu found that a constant intake of 3.7 g/day of dietary ALA may have biological effects similar to those of 0.3 g/day of dietary long chain n-3 PUFAs from fish oil. Eleven grams of ALA from flaxseed produces 1 g of the long chain n-3 PUFAs, EPA and DHA, and results in a calculated ratio of 14.8 g LA: 3.7 g ALA equivalent to a 4:1 ratio [61]. Supplementation with high amounts of ALA (>5.3 g/day) increased EPA plasma fatty acid and platelet concentrations [62,63]. Bjerve et al. noted that the minimum requirement for ALA in immobile adults was equivalent to 0.2% of total energy intake when n-3 long chain fatty acids were 0.08% of energy [64].

### Intake Suggestions to Meet the AI for n-3 and n-6 Fatty Acids

The recommendations for n-6:n-3 ratios suggested by experts and researchers, is in the range of 2-4:1 [5,44,57,58]. Other reviews regarding diet recommendations suggest an increase of ALA and a decrease of LA to meet the ratio or index [55,56]. If VGN intake of ALA increases, and LA is already at higher than recommended AI intakes, total essential PUFA intake may exceed the suggested intake of 1%–2% Kcals in the diet. If dietary LA amounts decrease dramatically, the g/day adult recommendation for ALA may also need to be decreased, in order to maintain a dietary n-6:n-3 ratio of around 4:1. Decreasing LA in the diet might be the best option if the VGN is overconsuming LA (more than 1–2% total dietary Kcals) or there are reasons why increased dietary ALA is not required (for example: already consuming ALA/EPA/DHA supplements, restrictive food or allergies/aversions). Decreasing dietary LA requires knowledge of good dietary sources of both LA and ALA, as well as the “hidden” sources of LA in processed foods, nuts, meat analogs, and salad dressings. Many salad dressings contain soybean oil, sunflower, safflower, or other oils high in LA [18]. Consuming less salad dressing or substituting high monounsaturated fatty acid nuts for nuts high in LA, could also be beneficial. Another strategy is to encourage VGNs to use high oleate sunflower oil instead of soybean or other high LA oils, since it contains high monounsaturated fats and low amounts of n-6 in salad dressing and other sauces to decrease LA intake. However, presently, there are no studies that have used this oil to decrease LA intake in VGNs, and it is suggested that more studies be done in this area of research.

Most nuts are high in LA and not very high in ALA, however, English walnuts are the exception, with values of 9 g of ALA and 38 g of LA per 100 g of chopped English walnuts, and a dietary n-6:n-3 ratio of 4:1.

Chia and flax (linseed) seeds are very high in ALA. Chia contains 17.8 g of ALA and 5.84 g of LA in 100 g, flaxseed contains 53.37 g of ALA and 14.25 g of LA, and hemp seeds contain 8.864 g of ALA and 1.34 g of ALA per 100 g [18] (Table 1). ALA supplements such as flax (linseed) oil, or marine algae supplements containing EPA and DHA, report less LA than other oils (see Table 1). Studies supplementing algal supplements show increases in serum and plasma EPA and/or DHA in vegetarians and VGNs [13,14,15]. However, until more studies are conducted that assess complete dietary intake of VGNs, including the lipid fractions that would contribute to biological n-3 and n-6 status as well as nutrients such as fiber with fat absorption and trans-fatty acids that interfere with elongation and desaturation, it is prudent not to suggest that VGNs take EPA and/or DHA supplements unless needed.

Davis and Kris Etherton suggest doubling the current AI recommendation of 1.1 g/day of ALA to 1.1 g/1000 cal or >1% of energy for n-3 fatty acids for VGNs and vegetarians on a 2000 Kcal diet, which may ensure intakes of at least 2.2 g/day (and up to 4.4 g/day) of ALA. This calculates to between 1%–2% Kcals of omega-3 fatty acids in the diet [54]. Simopoulous also stated that a ratio of 4:1 was associated with a 70% decrease in total mortality in secondary prevention of cardiovascular disease [56]. The recommendations and conclusions proposed by Davis and Kris Etherton suggest an increase of ALA of around 2.2–4.4 g/day depending on the amount of LA to meet a 4:1 ratio as well as a decrease of LA if intake of LA is higher than the AI. However, before decreasing dietary LA or increasing ALA, it is important to determine the intake of LA and ALA in the diet to determine whether ALA should be increased or LA decreased. VGNs, health professionals, researchers, or Registered Dietitian Nutritionists can assist by using computer software that contains comprehensive regularly updated research databases such as NDS-R Software (Regents of University of Minnesota) along with comprehensive client/patient diet records [65]. Some VGN diets are able to meet the AI for ALA as seen in Mann et al. [43] and Sanders et al. [46]. By decreasing LA in the diet and depending on the amounts of LA and the amount of ALA consumed, VGNs could potentially meet the present AI requirement, and permit ALA to utilize the elongation and desaturation enzymes without competitive inhibition from high somatic LA levels. Since endogenous conversion is needed for increased EPA and DHA somatic levels in VGNs, higher amounts than the AI for dietary ALA should be recommended for VGNs.

The data suggests increasing dietary recommendations for ALA to amounts higher than the present AI, maintaining dietary n-6:n-3 ratios of about 4:1 and less than 10:1 for VGNs, and possibly supplementing EPA and DHA with algal supplements for those individuals with increased need such as lactating and or pregnant VGNs, those lacking elongation or desaturase enzymes (inborne errors of metabolism).

## 7. Additional Research

Presently, the US n-3 and n-6 recommendations are based on the AI. Studies are needed to determine an EAR (used to set the RDA) and optimum ratios for both VGNs and omnivores across all population categories using dose response studies for both VGNs and non-VGNS.

### 7.1. Conversion Rates

There are differences in ALA conversion to EPA and DHA for males and females, and there is also a difference in ALA conversion by age [23,24,34] and in obese individuals [32]. More studies are needed to examine these differences in both VGNS and non-VGNs.

### 7.2. Diet and Biological Indicators

Studies should examine diet as well as biological indicators of the PUFA fractions, trans-fatty acids, and fiber (both soluble and insoluble), to determine VGN and omnivore tissue status.

Studies could be done to substitute high oleate sunflower oils in the VGN diet for high LA oils to determine if it is a viable way to decrease high LA intakes and influence n-3 and n-6 status.

#### 7.2.1. Biological Indicators and Tissue Status

Erythrocyte membranes may be a reliable indicator of long-term (3 or more months) essential fatty acid intake, but it is unknown whether calculated weights are as reliable as direct measurements. Adipose tissue is also a long-term indicator of essential fatty acid intake and/or conversion and can also be used to determine status.

More studies are needed to determine if serum and or plasma are reliable indicators of long-term n-6 and/or n-3 status.

Studies should also examine other n-3 and n-6 fatty acid fractions/biological indicators such as 18:3n-6 (6,9,12-octadecatrienioic-gamma linolenic acid-GLA), 20:3n-6 (8,11,14-eicosatrienoic acid-dihomo gamma linolenic acid-DGLA 20:4n-3 (8,11,14,17 eicosatetraenoic acid-ETA), 22:4n-6 (7,10,13,16-docosatetraenoic acid-adrenic acid), 22:5n-3 (7,10,13,16,19 docosapentaenoic acid-DPA), and the peroxisomals: 22:5n-6 (4,7,10,13,16-docosapentaeoic acid-osbond acid, 24:6n-3 (6,9,12,15,18,21-tetracosahexaenoic acid) and 24:5n-6 (6,9,12,15,18-tetracosapentaenoic acid). Identification of these long-chain fatty acids in biological indicators will help determine conversion status- especially in dose-dependent studies.

#### 7.2.2. Low n-3 Status/High n-6 Status and Risk

More research is needed to determine if low n-3 status and or high n-6 status leads to a risk of having a pro-inflammatory profile in both VGN and omnivores.

Burdge et al., suggested that there is a knowledge gap, and more studies of the effect of low DHA status due to maternal vegetarian or VGN diets should be done on the cognitive function in children [34].

More research is needed on the long-term effects of low erythrocyte and adipose EPA and DHA and chronic disease risk in both VGNs and omnivores.

## 8. Conclusions

The purpose of this review was to summarize relevant findings of studies on dietary intake and biological status of LA, AA, ALA, EPA, and DHA in adult, apparently healthy VGNs, and discuss the reasons why LA and ALA may need DRI Special Consideration requirements. We also provided dietary suggestions as to how VGNs can ensure adequate intakes of LA and ALA.

To summarize, the present DRI AI for essential fatty acids is intended for general healthy populations, and does not take into account groups with specific dietary needs/requirements. We suggest that adult VGNs have separate AIs for LA and ALA than omnivores and utilize recommendations of between 2.2–4.4 g/g of ALA d (or 1.1 g/day/1000 Kcals). Additionally, a 4:1 n-6:n-3 ratio could be considered for both VGNs and omnivores, since there are no present recommendations for an essential fatty acid ratio.

It is advisable for VGNs to decrease unnecessary or high sources of LA in the VGN diet, especially if they consume ≥10:1 ratio of n-6:n-3, and/or greater than the AI for LA. Decreasing high LA intake in VGNs could be an option; however, decreasing LA intake without being mindful of ALA dietary sources that are also high sources of LA might also decrease ALA intake. In order to meet the AI for LA and ALA, it is suggested that VGNs and health professionals become aware of the amounts of LA and ALA (and possibly trans-fatty acid) in VGN intakes, encourage consumption of food sources for each (high food sources provided in Table 1) and make dietary additions/corrections to the VGN diets as needed.

## 9. Main Findings from This Review

1. Most studies indicate that VGNs consume higher amounts of LA compared to omnivores, with confirmation in tissues stores; however, there are inconsistent findings of AA tissue concentrations compared to omnivores.
2. There are inconsistent results of ALA intake by VGNs compared to omnivores.
3. Most studies show that VGNs consume low to zero amounts of EPA and DHA, unless they take supplements.
4. Most studies indicate that plasma, serum, erythrocytes, adipose, and platelet levels of EPA and DHA are lower in VGNs than omnivores.
5. VGNs may need an ALA increase of 2.2–4.4 g/day (or 1.1 g/day/1000 Kcals) depending on the amount of LA in the diet in order to achieve a 4:1 n-6:n-3 ratio, as well as a decrease of dietary LA if intake of LA is higher than recommended.
6. Special consideration recommendations for both ALA and LA for adult VGNs should be considered by the AI/DRI.

## Figures and Tables

**Table 1:** Energy, protein, total lipid, 18:2 n-6, 18:3 n-3, carbohydrate, and fiber contents of oils and foods commonly consumed by vegans and vegetarians for the essential fatty acids.

Nutrient ^1^		Energy	Protein	Total Lipid	18:2n-6	18:3n-3	CHO	Total Fiber
	Unit	Kcal	g	g	g	g	g	g
Oils								
Oil, Canola	100 g	884	0	100	18.64	9.137	0	0
Oil, Flaxseed/Linseed (Panos)	100 g	884	0.110	99.98	14.25	53.37	0	0
Oil, Soybean	100 g	763	0	100	50.42	6.789	0	0
Oil, Walnut	100 g	884	0	100	52.90	10.40	0	0
Oil, High Oleic Sunflower	100 g	884	0	100	3.61 *	0.192 **	0	0
Oil, High Oleic Safflower	100 g	884	0	100	12.72	0.096	0	0
Oil, Culinary Algae	100 g	800	0	93.33	n/a	n/a	0	0
Ovega-3 supplement ^2^	1 gel	n/a	0	n/a	n/a	0.5	0	0
Deva Vegan supplement ^3^	1 cap	5	0	0.5	n/a	0.2	0	0
Nordic Naturals supplement ^4^	2 gels	10.0	0	1.00	n/a	0.715	0	0
Food Sources								
Almonds Raw	100 g	579	21.15	49.93	12.30	0.003	21.55	12.50
Amaranth	100 g	371	13.65	7.020	2.736	0.042	65.25	6.70
Avocados, Raw, California	100 g	167	1.960	15.41	1.674	0.111	8.640	6.80
Black Walnuts Dried	100 g	619	24.06	59.33	33.80	2.680	9.580	6.80
Brazil Nuts, Dried	100 g	659	14.32	67.10	23.859	0.018	11.74	7.50
Brown Rice Cooked	100 g	123	2.740	0.970	0.355	0.011	25.58	1.60
Bulgur Cooked	100 g	83	3.08	0.240	0.094	0.004	18.58	4.50
Cashews Raw	100 g	553	18.22	43.85	7.782	0.062	30.19	3.30
Chia Seeds Dried	100 g	486	16.54	30.74	5.840	17.80	42.12	34.4
English Walnuts Dried	100 g	654	15.23	65.21	38.09	9.08	13.71	6.70
Flaxseed Raw	100 g	534	18.29	42.16	5.903	22.81	28.88	27.3
Hempseed Hulled	100 g	553	31.56	48.75	1.340	8.864	8.670	4.00
Millet Cooked	100 g	119	3.510	1.000	0.480	0.028	23.67	1.300
Oat Bran Cooked	100 g	40	3.210	0.860	0.324	0.015	11.44	2.60
Pistachio Raw	100 g	560	20.16	45.32	13.10	0.210	27.17	10.60
Poppy Seeds	100 g	525	17.99	41.56	28.30	0.273	28.13	19.50
Quinoa	100 g	368	14.12	6.070	2.977	0.260	64.16	7.00
Rye	100 g	338	10.34	1.630	0.659	0.108	75.86	15.1
Sesame Seeds dried	100 g	573	17.73	49.67	21.375	0.376	23.45	11.8
Soybeans Raw	100 g	446	36.49	19.94	9.925	1.330	30.16	9.30
Soybeans, Boiled	100 g	141	12.35	6.400	2.657	0.354	11.05	4.20
Sunflower Seeds	100 g	584	20.78	51.50	23.05 *	0.06 **	20.00	8.60

^1^ Source: US Department of Agriculture Nutrient Data Base, 2019 [18]. * undifferentiated 18:2, ** undifferentiated 18:3. ^2^ Ovega-3 i-Health, Inc., 2017 [19]. ^3^ Deva Nutrition LLC, 2017 [20]. ^4^ Nordic Naturals Inc., 2017 [21].

**Table 2:** Concentrations of n-6 fatty acids in various tissues by following a vegan or non-vegan diet (as cited in text).

Author, Year	Plasma	Serum	Erythrocyte and Whole Blood	Platelets	Breastmilk	Adipose
Rosell et al., 2005 [10]	Higher concentrations of LA in VGN plasma fatty acids than in meat-eaters, but found no significant differences in AA.	-	-	-	-	-
Li et al., 1999 [39]	Higher concentrations of LA in VGN plasma fatty acids than in meat-eaters, but found no significant differences in AA.	-	-	VGNs had higher levels of LA in platelets, but lower levels of AA than the high meat-eaters.	-	-
Mann et al., 2006 [45]	Higher concentrations of LA in VGN plasma fatty acids than in meat-eaters, but found no significant differences in AA.	-	-	-	-	-
Pinto et al., 2017 [9]	No significant differences between plasma LA or AA in VGNs or omnivores.	-	No difference for LA or AA in blood fatty acid profiles of VGNs and meat eaters.	-	-	-
Sanders et al., 1978 [46]	Higher LA and AA plasma choline phospholipids in VGNs compared to omnivores.	-	Higher levels of LA in VGNs than omnivores, but no significant differences for AA in erythrocytes. LA was higher in breastfed VGN infant erythrocytes compared to omnivore breast-fed infants, and AA was not significantly different.	-	-	Higher levels of LA in VGN adipose tissue compared to omnivore adipose tissue.
Elorinne et al., 2016 [11]	-	Higher LA, but no significant differences of AA in VGN serum compared to non-VGN serum.	-	-	-	-
Agren et al., 1995 [42]	-	Higher LA, but no significant differences of AA in VGN serum compared to non-VGN serum.	Higher levels of LA in VGNs than omnivores, and higher AA in omnivores.	Higher levels of LA in VGN platelets, but no differences of AA compared to omnivores.	-	-
Sarter et al., 2015 [16]	-	-	No difference for LA or AA in blood fatty acid profiles of VGNs and soldiers.	-	-	-
Kornsteiner et al., 2008 [40]	-	-	Higher levels of LA in VGNs than omnivores, and higher AA in omnivores.	-	-	-
Fisher et al., 1986 [47]	-	-	-	Higher platelet LA concentration, and lower AA in both the VGN and vegetarian subgroups as compared to omnivores.	-	-
Sanders et al., 1992 [48]	-	-	-	-	Higher levels of LA in VGN breastmilk compared to omnivores, but there were no significant differences for AA.	-
Perrin et al., 2018 [12]	-	-	-	-	No significant differences for LA or AA in VGNs as compared to omnivores. LA:ALA ratio was significantly lower in VGNs than omnivores.	-
Miles et al., 2019 [41]	-	-	-	-	-	LA was higher in VGNs than non-vegetarians. AA was significantly lower in VGNs than non-vegetarians.

**Table 3:** Concentrations of n-3 fatty acids in various tissues by following a vegan or non-vegan diet (as cited in text).

Author, Year	Plasma	Serum	Erythrocyte and Whole Blood	Platelets	Breastmilk	Adipose
Rosell et al., 2005 [10]	Plasma ALA was not different between male VGNs and meat-eaters, whereas EPA and DHA were significantly lower in male VGNs than in meat-eaters.	-	-	-	-	-
Mann et al., 2006 [45]	ALA plasma was higher in male VGNs than the high meat group, whereas EPA and DHA were significantly lower in VGNs.	-	-	-	-	-
Agren et al., 1995 [42]	Lower levels of DHA and EPA in VGNs compared to omnivores.	Lower levels of DHA and EPA in VGN serum compared to omnivores.	DHA and EPA were lower in VGN erythrocytes compared to omnivores.	Low levels of DHA and EPA in VGN platelets compared to omnivores.	-	-
Sanders et al., 1978 [46]	EPA and DHA were significantly lower compared to omnivores.	-	Low EPA and DHA in VGNs and VGN breast-fed infant erythrocytes compared to omnivores.	-	Higher ALA levels in VGNs than omnivores, and lower DHA and EPA (not significant).	ALA was higher in adipose tissue of VGNs than omnivores (not significant).
Li et al., 1999 [39]	EPA and DHA were lower in male VGNs, and the n-3:n-6 ratio was lower, and AA:EPA was higher compared to male high meat eaters.	-	-	No differences in ALA composition between VGNs and omnivores; however, there was a significant decrease in EPA and DHA levels in VGNs.	-	-
Pinto et al., 2017 [9]	ALA was higher in VGNs than the high meat group; however, EPA and DHA were significantly lower in VGNs.	-	The omega-3 index was significantly lower in VGNs (2.71%) than in omnivores (5.42%).	-	-	-
Welch et al., 2010 [44]	ALA levels were not different for males or females as compared to the meat-eaters, but VGN DHA was higher in women than meat-eaters. In VGN males, plasma DHA was lower than omnivores.	-	-	-	-	-
Elorinne et al., 2016 [11]	-	No differences between VGNs and non-VGN serum ALA concentrations, however EPA and DHA serum concentrations were significantly lower in VGNs than omnivores.	-	-	-	-
Sanders et al., 1992 [48]	-	-	The proportion of DHA in erythrocytes of breast-fed VGN infants was 1.9% as compared to infants fed a milk formula (3.7%).	-	Levels of ALA were comparable to vegetarians, but concentrations of DHA were less than 50% for both vegetarians and omnivores.	-
Kornsteiner et al., 2008 [40]	-	-	DHA and EPA in adult erythrocytes were decreased in Austrian VGNs.	-	-	-
Sarter et al., 2015 [16]	-	-	Higher ALA and EPA levels in VGNs. No differences in DHA levels between VGNs and US soldiers in whole blood FA.	-	-	-
Sanders et al., 1992 [22]	-	-	-	No difference in ALA composition between VGNs and non-VGNs; however, there was a sig. decrease in EPA and DHA levels in VGNs.	-	-
Perrin et al., 2018 [12]	-	-	-	ALA was significantly higher in VGNs than in non-VGNs; DHA and EPA were not sig. different. The LA:ALA ratio was lower in VGNs than non-VGNs.	-	-
Miles et al., 2019 [41]	-	-	-	-	-	ALA was higher and EPA, DPA, and DHA were sig. lower in VGNs than non-VGNs. Total n-3 was highest in VGNs due to high ALA levels.

**Table 4:** Recommendations by researchers and reviewers for adult vegans/total vegetarians (VGNs) (most recent studies first).

Author, Year	Recommendation: Males; Females Combined	18:2n-6 LA	18:3 n-3 ALA	20:5 n-3 EPA	22:6 n-3 DHA	n-6:n-3 or Omega-3 Index
Agnoli et al., 2017 [55]	Females	Limit intake of sources of n-6 FA, & TFA. Limit consumption of processed, deep-fried foods, and alcohol.	-	Pregnant/breastfeeding or women with increased requirement for long chain n-3 fatty acids, should be advised to consume an algae-based supplement of known nutrient content.	Pregnant/breastfeeding or women with increased requirement for long chain n-3 fatty acids, should be advised to consume an algae-based supplement of known nutrient content.	-
	Combined-Males and Females	-	Improve n-3 nutritional status by regular consumption of good sources of alpha-linolenic acid.	-	-	-
Burdge et al., 2017 [34]	Females	-	-	-	Need more cognitive studies for children of vegetarian females due to low DHA levels.	-
Pinto et al., 2017 [9]	Combined	-	Further research whether populations with low n-3 status are more at risk of having a pro-inflammatory profile.	-	-	-
Melina et al., 2016 [5]	Females	-	-	-	Low dose microalgae DHA supplements for pregnancy and lactation.	-
	Combined	High intakes of LA may suppress ALA conversion.	May be prudent to ensure higher intakes of ALA. N-3 needs of healthy individuals can be met with ALA alone; endogenous synthesis of EPA and DHA from ALA is sufficient.	Clinical relevance of reduced EPA and DHA status in VGNs are unknown. Low-dose micro-algae based DHA supplements are available for those with increased needs.	Clinical relevance of reduced EPA and DHA status in VGNs are unknown. Low-dose micro-algae based DHA supplements are available for those with increased needs.	A ratio of LA/ALA not exceeding 4:1 has been suggested for optimal conversion.
Harris, 2014 [56]	Combined	Consume more ALA and less LA to increase the Omega-3 Index.	Consume more ALA and less LA to increase the Omega-3 Index.	-	-	Omega-3 Index >8%.
Sarter et al., 2014 [16]	Combined	-	-	VGNs respond robustly to a relatively low dose of a vegetarian omega-3 supplement.	VGNs respond robustly to a relatively low dose of a vegetarian omega-3 supplement.	No direct evidence that omega-3 index confers additional health benefits over and above their already protective VGN diet.
Saunders et al., 2013 [15]	Combined	-	Double current AI of ALA if no direct sources of EPA and DHA are consumed. Increased needs or reduced conversion may benefit from DHA and EPA supplements derived from microalgae of 200–300 mg/day of DHA and EPA (pregnant and lactating women) and reduced conversion ability.	-	-	-
Craig, 2010; Review [51]	Combined	-	Regular consumption of plant foods naturally rich in n-3 fatty acid ALA, such as ground flaxseed, walnuts, soy products, and hemp seed beverages.	-	Pregnant and lactating women may benefit from DHA-fortified foods and microalgae-derived DHA supplements.	-
Welch et al., 2010 [44]	Combined	-	Further research for conversion of ALA to long chain n-3 PUFAs for maintenance of adequate status in non-fish and fish-oil consumers is required.	-	-	-
Simopoulos, 2008 [57]	Combined	-	-	-	-	Ratio of 4:1 is associated with 70% decrease in total mortality in secondary prevention of cardiovascular disease.
Kornsteiner et al., 2008 [40]	Combined	-	Erythrocyte phospholipid n-3 status of VGNs is critical. It is important to maintain n-3 fatty acid intake during adult life.	-	-	Ensure physical, mental and neurological health, reduce n-6/n-3 ratio with an additional intake of direct sources of EPA and DHA, regardless of age and gender.
Mann et al., 2006 and Li et al., 1999 (Mann used data collected from Li, 1999) [45]	Combined	-	Advised to increase intake of n-3 fatty acids to increase platelet PL n-3 PUFA and reduce platelet aggregability.	-	-	-
Rosell et al., 2005 [10]	Combined	-	Suggests (with caution) VGNs increase intake of ALA and limit intake of LA to optimize FA status. The importance of long chain fatty acids in the diet needs further investigation.	-	-	-
Davis et al., 2003 [53]	Combined	5.5–8% Kcals from n-6 FA. Reduce high n-6 oils in the diet, decrease processed foods. Primary fat should be MUFA.	<1.5–2% of calories should be obtained from n-3. Decrease EtOH and trans fatty acids to increase conversion of ALA to EPA and DHA. Double intake of ALA; provide >1% of energy from n-3 or about 1.1 g/1000 calories. Increased needs require 2.2 g/1000 calories. Aim for 2–4 g of ALA/day.	Not essential, but it is important to ensure sufficient levels by relying on conversion from parent fatty acids. Two important steps to improve EPA status: 1) Maximize conversion of ALA to EPA and DHA and 2) Provide a direct source of EPA and DHA.	Not technically essential, but ensure sufficient levels by relying on conversion from parent fatty acids. Supplements of DHA should be 100–300 mg/day. DHA and ALA should also be adequate.	To achieve the 4:1 ratio, <1.5–2% of calories should be obtained from n-3 and 5.5–8% of calories from n-6 FA.
Simopoulos, 2000 [54]	Combined	-	-	-	-	Ratio was 1:1 to 2:1 in ancient diets. Present ratio is 10 to 1:20 to 25 to 1- indicates a deficiency in Western Diets of n-3.
Fokkema et al., 2000 [43]	Combined	-	-	-	-	Found a 3:1 ratio increased EPA from 0.3 to about 1.0% in VGN plasma. A 3.7 g/day ALA diet for 4 weeks (ratio of 3.8:1) augmented ALA about 1% more. Total n-3 levels increased from 2.3 to 3.4% in plasma. DHA status was not increased.
Agren et al., 1995 [42]	Combined	Depressed levels of n-3 FA are depressed due to very low diet levels and high LA and oleic acid intake.	-	-	-	-
Sanders et al., 1978 [46]	Females	-	More research is needed on low delta 4 desaturase in lactating women.	-	-	-
	Combined	-	Further research needed to establish whether differences in the proportion of n-3 derivatives in tissues are of physiological importance.	-	-	-
